# Engineering Protein–Peptide Interfaces via Combinatorial Mutagenesis and Mass Photometric Screening

**DOI:** 10.3390/biom15081183

**Published:** 2025-08-18

**Authors:** Bitasadat Hosseini, Mohammed Ashraf, Philip Kitchen, Anupama Chembath, Russell Collighan, Corinne M. Spickett, Lynne Regan, Anna V. Hine

**Affiliations:** 1 School of Biosciences, College of Health and Life Sciences, Aston University, Aston Triangle, Birmingham B4 7ET, UK; 200218289@aston.ac.uk (B.H.); m.ashraf2@aston.ac.uk (M.A.); p.kitchen1@aston.ac.uk (P.K.); a.chembath1@aston.ac.uk (A.C.); r.collighan@aston.ac.uk (R.C.); c.m.spickett@aston.ac.uk (C.M.S.); 2 Aston Institute for Membrane Excellence (AIME), Aston University, Aston Triangle, Birmingham B4 7ET, UK; 3 Centre for Synthetic & Systems Biology, Institute for Quantitative Biology, Biochemistry and Biotechnology, School of Biological Sciences, University of Edinburgh, The King’s Buildings, Edinburgh EH9 3JU, UK; lynne.regan@ed.ac.uk

**Keywords:** SpyTag–SpyCatcher, combinatorial libraries, MAX randomization, positional saturation mutagenesis, overlap PCR, mass photometry

## Abstract

The SpyTag–SpyCatcher system, developed by the Howarth lab, is based on splitting the CnaB2 domain from Streptococcus pyogenes into two parts: a 13-amino-acid SpyTag and a 116-amino-acid SpyCatcher. Upon incubation, they spontaneously form a covalent isopeptide bond between Asp7 (SpyTag) and Lys31 (SpyCatcher). This study explores whether the interaction specificity can be modulated by altering hydrophobic residues within the SpyCatcher binding pocket and corresponding SpyTag positions, potentially to create orthogonal SpyTag–SpyCatcher pairs. Libraries of SpyCatcher and SpyTag were created by partial saturation mutagenesis using overlap PCR and MAX randomisation, respectively. To assess the specificity of the SpyCatcher–SpyTag interaction within the resulting protein mixtures, a novel screening strategy based on mass photometry was developed to detect isopeptide bond formation. We demonstrate tolerance to mutation in the hydrophobic binding pocket of SpyCatcher in terms of binding native SpyTag and demonstrate what to our knowledge constitutes the first example of using mass photometry to examine the interactions of small libraries of proteins with a given ligand. Mass photometry detects stable interactions whether covalent or not and so this study suggests the prospect of employing mass photometry for more general application in protein engineering.

## 1. Introduction

The ability to link proteins together in a site-specific, stable, and covalent manner under physiological conditions is central to numerous applications in biotechnology, synthetic biology, and biomedicine. Among the available protein ligation tools, the SpyTag–SpyCatcher system has emerged as a particularly attractive platform. This system, engineered from the Streptococcus pyogenes fibronectin-binding protein FbaB, consists of a short peptide tag (SpyTag) and a protein partner (SpyCatcher), which spontaneously form an intramolecular isopeptide bond between defined lysine and aspartate residues [1,2,3]. The reaction is irreversible, highly specific, and operates efficiently in diverse environments, which has enabled its adoption in protein cyclisation, surface immobilisation, vaccine design, and hydrogel formation [4,5,6,7].

Efforts to enhance this platform have focused primarily on improving ligation kinetics through rational design. Notably, the development of SpyCatcher002–SpyTag002 and SpyCatcher003–SpyTag003 has led to increased reaction rates, greatly expanding the system’s utility for dynamic intracellular processes [8,9]. However, despite these advances, the field still lacks a general strategy for engineering new SpyCatcher variants with distinct or orthogonal binding specificity. Most current studies retain the original peptide–protein interface, and little is known about the sequence constraints that govern specificity in this system. This limitation restricts the development of modular protein architectures.

Protein engineering strategies based on saturation mutagenesis and combinatorial libraries offer a powerful means to overcome this limitation. Combinatorial libraries, particularly those with fixed positions, have long been employed in enzymology and drug discovery to map binding preferences and design new functional interfaces [10,11]. Yet such approaches are rarely used to engineer covalent protein–peptide interactions. Here, we generate a series of SpyCatcher variants via non-degenerate saturation mutagenesis at key hydrophobic residues within the peptide-binding cleft of SpyCatcher, and interrogate the specificity of resulting SpyCatcher variants using positionally fixed SpyTag peptide libraries. The use of non-degenerate codons ensures efficient exploration of sequence space while avoiding issues such as codon redundancy or premature termination codons [12].

A central innovation of this work lies in its screening strategy: we employ mass photometry, a label-free, relatively recent, single-molecule technique capable of detecting molecular interactions and complex stoichiometries in solution with high precision [13,14]. Mass photometry employs interferometric scattering microscopy, as described by Young et al. [15]. In essence, laser light is shone on a small volume of the molecule(s) of interest on a glass cover slip. That light is either reflected by the glass or is scattered by molecules in contact with the glass (termed a “landing event” [16]). Scattered light interferes with the reflected light in a manner that is proportional to the molecular mass of the molecule(s) concerned [16,17] and subsequent image analysis determines contrast—the ratio of a signal resulting from each landing event to the signal generated by reflection from the coverslip itself [16]. The technology is the subject of several excellent, recent reviews [18,19,20] and can be used to measure any molecular interaction—whether covalent or non-covalent, providing that the mass of the molecule(s) concerned exceeds a lower detection limit, which is typically around 30 kDa [17,18]. Unlike traditional display-based selections, which rely on biopanning of displayed libraries and subsequent sequence elucidation, mass photometry thus enables rapid and quantitative analysis of binding events without requiring labelling, immobilisation or any type of link between phenotype and genotype.

There is an urgent need for modular bioconjugation systems that combine specificity, covalency, and orthogonality. The combination of rational mutagenesis, chemically defined libraries, and real-time phenotypic screening permits mapping of sequence–function relationships in the SpyTag–SpyCatcher system. Expanding the SpyTag–SpyCatcher toolbox to include new binding profiles would open the door to multiplexed tagging schemes, programmable assembly of protein scaffolds, targeted delivery vehicles, and synthetic biological circuits with multiple independent ligation events. While the core concept of engineering covalent protein–peptide interactions is well established, the development of orthogonal variants using systematic, library-based approaches remains underexplored.

The aim of this study was to investigate whether the binding specificity of the SpyTag–SpyCatcher system could be altered to generate orthogonal interaction pairs through targeted mutagenesis. While previous work has successfully enhanced the reaction kinetics of this system [8,9], few efforts have explored modifications that confer new or selective binding preferences. Instead, applications requiring controlled conjugation of multiple proteins have relied on combining distinct systems such as SpyTag–SpyCatcher and SnoopTag–SnoopCatcher [21].

Here, guided by molecular modelling, we focused on substituting three hydrophobic residues within the SpyCatcher hydrophobic pocket (I27, M44 and I90) and two corresponding positions within SpyTag (I3 and M5), substituting each with a defined set of hydrophobic amino acids (I, L, V, M, F, Y). Our objective was to determine whether such substitutions could disrupt, preserve, or reprogramme binding specificity, with the potential to produce orthogonal pairs. Accordingly, we designed and screened targeted SpyCatcher variant libraries against native and combinatorial SpyTag libraries using mass photometry, enabling direct, quantitative or qualitative detection of binding events in solution.

## 2. Materials and Methods

Library synthesis, cloning, sequence verification, expression and purification. SpyCatcher libraries were generated by first amplifying PCR fragments encompassing codons 27 and 44 and separately, 44 and 90 of SpyCatcher, using the mutagenic primers listed in Supplementary Table S1. Various pairs of these PCR products were then joined by overlap PCR to create the required SpyCatcher libraries and the resulting products were purified using a DNA Clean & Concentrator-5 kit according to manufacturer’s instructions (Zymo Research, Irvine, CA, USA, cat D4013). SpyTag libraries were created by MAX randomisation as previously described [22,23], from oligonucleotides listed in Supplementary Table S2. Each resulting library (both SpyCatcher and SpyTag libraries) were amplified with Golden Gate primers (Supplementary Tables S1 and S2) and joined to similarly amplified pET vectors via Golden Gate assembly (New England Biolabs, Ipswich, MA, USA, cat. E1601S). Resulting products were transformed into E. coli DH5α cells and plasmid libraries purified directly from liquid culture. Resulting plasmid libraries were assessed by NGS sequencing (Amplicon EZ service, Genewiz by Azenta Life Sciences, Oxford, UK) and/or by Sanger sequencing (University of Birmingham, Functional Genomics Laboratory, Birmingham, UK) as indicated. NGS data were interpreted as described previously [23]. Plasmid libraries were subsequently transformed into E. coli Tuner (DE3) cells (Novagen—Merck, UK, cat 70623) and incubated in LB with shaking at 37 °C. Once an O.D A600 of 0.5 had been reached, expression was induced by adding IPTG to a final concentration of 1 mM. Thereafter, the cells were incubated overnight with shaking at 20 °C. Cells were harvested by centrifugation and lysed using BugBuster protein extraction reagent (Merck, UK, cat 70584-M) according to manufacturer’s instructions. Lysates were clarified by centrifugation (16,000× *g*, 20 min, 4 °C) and either used directly or stored at −20 °C. His-tagged protein libraries were bound to Ni^2+^-NTA resin (HisPur™ Ni-NTA Resin, Thermo Scientific, Abingdon, UK, cat. 88221), washed with imidazole-containing buffer, and eluted with 250 mM imidazole. Eluates were analysed by SDS-PAGE and protein concentration was determined using the BCA assay.

SpyTag/SpyCatcher binding reactions. To assess peptide–protein binding, mixtures (10–20 μM) of SpyTag and SpyCatcher variants were incubated in PBS (pH 7.4) at 25 °C for 3 h at 1:1 (individual SpyCatcher–SpyTag reactions), 1:6 (SpyCatcher–SpyTag library reactions) or 1:18 (SpyCatcher library–SpyTag reactions) molar ratios. Binding was analysed by SDS-PAGE or mass photometry.

SDS-PAGE analysis. SDS-PAGE was performed on 12% polyacrylamide gels using Mini-PROTEAN^®^ (Bio-Rad, Hercules, CA, USA) units. Samples were mixed with Laemmli buffer, denatured at 95 °C for 7 min, and electrophoresed at 18 V/cm. Resulting gels were photographed with a digital camera under UV illumination.

Mass photometry. Mass photometry was conducted using a Refeyn MPTwo instrument according to the manufacturer’s instructions. In brief, fresh PBS pH 7.4 (Thermo Scientific, cat. 003002) was prepared and filtered through 0.22 μm filters. Proteins or protein mixtures were diluted to 500–2000 nM and calibration was performed using NativeMark (ThermoFisher; diluted 1:20 in PBS). A drop of immersion oil was placed on the lens and a cleaned coverslip and gasket mounted. Each sample (2 μL) was added to 18 μL buffer on the coverslip to give a final concentration of 50–200 nM, and data acquired over 60 s. Calibration files were loaded and contrast-to-mass plots were generated in the Refeyn DiscoverMP software, version 2024 R2.1. Mass histograms were adjusted for bin size prior to export.

## 3. Results

### 3.1. Library Design

To explore the specificity of the hydrophobic, elements of the SpyCatcher–SpyTag interaction and whether there is potential for engineering orthogonal SpyCatcher–SpyTag pairs, three hydrophobic residues within SpyCatcher (positions 27, 44, and 90) and two corresponding residues of SpyTag (positions 3 and 5) were selected for limited saturation mutagenesis. These selections were based on Howarth and co-workers’ prior structural analysis of the SpyCatcher/SpyTag interaction, which identified that residues Ile27, Phe29, Met44, Phe75, Ile90 and Phe92 form a hydrophobic binding cleft into which residues Ile3 and Met5 of SpyTag are inserted [2]. Howarth and co-workers further describe hydrogen bonding and electrostatic interactions involving residues Glu34, Asp35, a water molecule and Gly83, Glu85 (and potentially, Lys37) with residues Tyr9 and Lys 10 of SpyTag both via side chain and the peptide backbone interactions [2]. Collectively these structures provide the microenvironment in which isopeptide bond formation between residue Lys31 of SpyCatcher and Asp7 of SpyTag occurs, involving catalytic residue Glu77 [3,24].

We wished to avoid perturbing the polar environment involved with isopeptide bond formation, we elected to study the hydrophobic residues in both the peptide-binding cleft of SpyCatcher and/or the corresponding hydrophobic residues of SpyTag which are inserted into that cleft, and selected corresponding aliphatic residues to determine whether or not it would be possible, buy changing these residues, to generate orthogonal SpyCatcher/SpyTag pairs. Accordingly, the hydrophobic amino acids valine, isoleucine, leucine, methionine, phenylalanine were selected for substitution at each of the three identified positions. Tyrosine was additionally selected to evaluate whether a little polarity might also be tolerated within the interaction. Thus, in total, 216 (6 × 6 × 6) SpyCatcher mutants were envisaged using the same six-residue set, yielding 36 (6 × 6) unique SpyTag variants.

**Figure 1:**
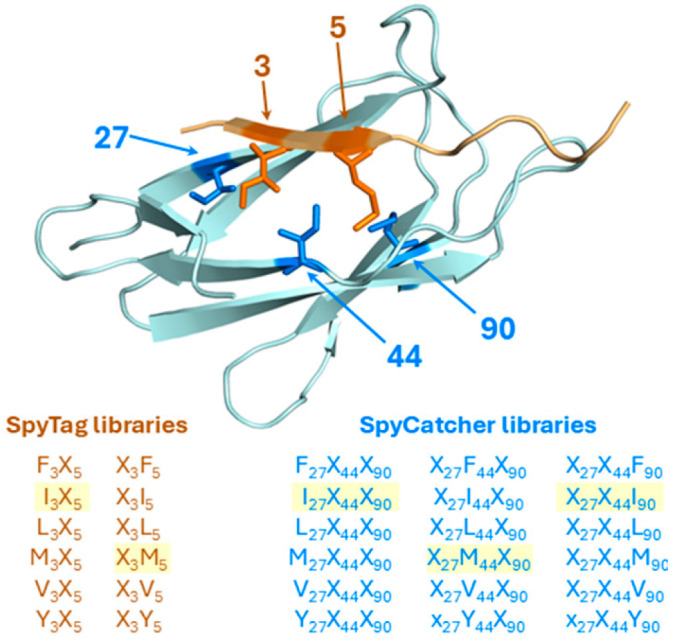
Top: cartoon representation of the SpyCatcher–SpyTag interaction highlighting residues selected for library generation. The image was generated from PDB 4MLI using PyMol (Schrödinger, Inc., New York, USA). SpyCatcher is represented in blue, while SpyCatcher is represented in orange. Bottom: identities of positions and composition of positionally fixed SpyTag (orange) and SpyCatcher (blue) libraries. Libraries that contain the native SpyTag or SpyCatcher are highlighted in yellow.

While generating and screening all 7776 (216 × 36) potential SpyCatcher–SpyTag combinations would be experimentally impractical, a positional fixing strategy inspired by Houghten’s combinatorial approach was implemented [9]. In essence, the SpyCatcher library was subdivided into 18 smaller libraries, each encoding 36 variants, arranged into three groups where one of the three positions (27, 44, or 90) was of fixed identity, while the remaining two positions were randomised to encode all six selected amino acids. Separately, small libraries of the SpyTag, similarly fixed/randomised at positions 3 and 5 were also designed to enable targeted, efficient screening against SpyTag variants while maintaining combinatorial diversity across the SpyCatcher interface. The concept is illustrated in Figure 1.

### 3.2. Library Synthesis

Owing to the distance between the selected codons within the SpyCatcher gene, overlap PCR was chosen as the technique to introduce targeted mutations into the 18 positionally fixed SpyCatcher libraries illustrated in Figure 1. Conversely, the proximity of targeted codons allowed the 12 SpyTag libraries to be synthesised by MAX Randomisation [22]. SpyCatcher DNA libraries were cloned as fusions to mNeonGreen while SpyTag libraries were cloned as fusions to mCherry. Having established an acceptable codon distribution in the randomised positions (Supplementary Figure S1), each library was expressed in *E. coli* and the resulting protein mixtures were isolated by affinity chromatography. These fusions were chosen both to allow flexibility in subsequent assays and to make the products large enough to be examined by mass photometry, which has a lower cut-off of ~30 kDa.

### 3.3. Analysis of the Native SpyCatcher–SpyTag Interaction by Mass Photometry

Mass photometry enables the label-free detection of molecular interactions. For this type of assay, the presence of fluorescent protein tags is irrelevant other than in terms of their molecular mass. To evaluate the suitability of this technique for analysing the SpyCatcher–SpyTag interaction, native SpyCatcher-mNeonGreen (42 kDa, hereafter “SpyCatcher”), native SpyTag-mCherry (30 kDa, hereafter “SpyTag”) and a 1:1 molar mixture of the two were initially assessed by mass photometry.

**Figure 2:**
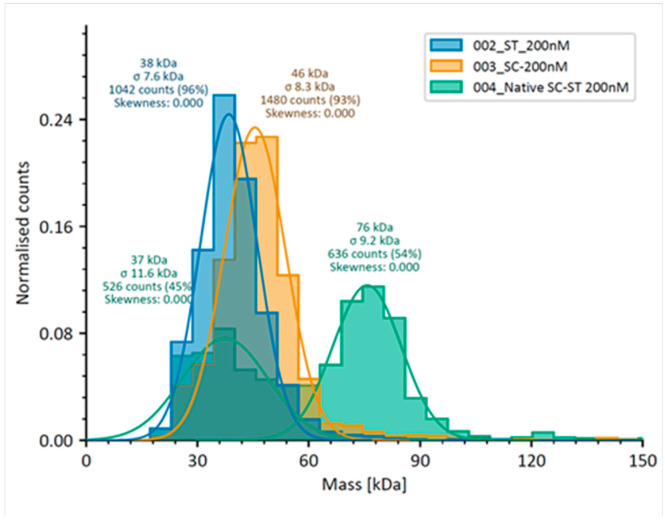
Mass photometry analysis of native SpyTag peptide (ST_200nM, blue), native SpyCatcher protein (SC_200nM, orange) and the product of their interaction (Native SC-ST_200nM, green). Each dataset represents normalised counts. Results have been overlayed to permit comparison.

As illustrated in Figure 2, which represents an overlay of three different spectra (Spy Catcher, SpyTag and the SpyCatcher–SpyTag interaction), mass photometry was indeed able to detect both the component Spy Catcher and SpyTag proteins and the covalently bonded product that results from their interaction. In such a case, mass photometry analysis is quantitative. SpyTag alone (ST_200nM, blue) gave a peak at 38 kDa with a standard deviation (σ) of 7.6 kDa and 1042 counts (actual MW = 30 kDa) and representing 96% of the sample. SpyCatcher alone (SC_200nM, orange), exhibited a peak at 46 kDa (σ = 8.3 kDa) with 1480 counts, accounting for 93% of the sample, consistent with the expected molecular weight of the SpyCatcher protein at 42 kDa [25] and suggesting a slight over-estimation of molecular weight by the technique, which is unsurprising as these molecular weights are both very close to the lower limit of detection for mass photometry, and smaller than the lowest mass point on the calibration curve (66 kDa). Following the incubation of SpyTag with SpyCatcher, a new peak (Native SC-ST_200nM, green) was evident at 76 kDa (σ = 9.2 kDa), with 636 counts representing 54% of the sample and corresponding to the anticipated 72 kDa covalent SpyTag–SpyCatcher product, whilst the remaining material in this sample (37 kDa, 526 counts, 45%) represents a combination of unreacted SpyTag and SpyCatcher fusion proteins. Collectively, the covalently bonded and individual components of the reaction comprise 99% (54% + 45%) of detected molecules. In summary, the mass shift from the individual components to the new peak indicated successful bonding between the two molecules, leading to the formation of a stable covalent complex with a mass approximately equivalent to the sum of SpyTag and SpyCatcher [25].

### 3.4. Many Hydrophobic Variants of SpyCatcher Can React with Native SpyTag

**Figure 3:**
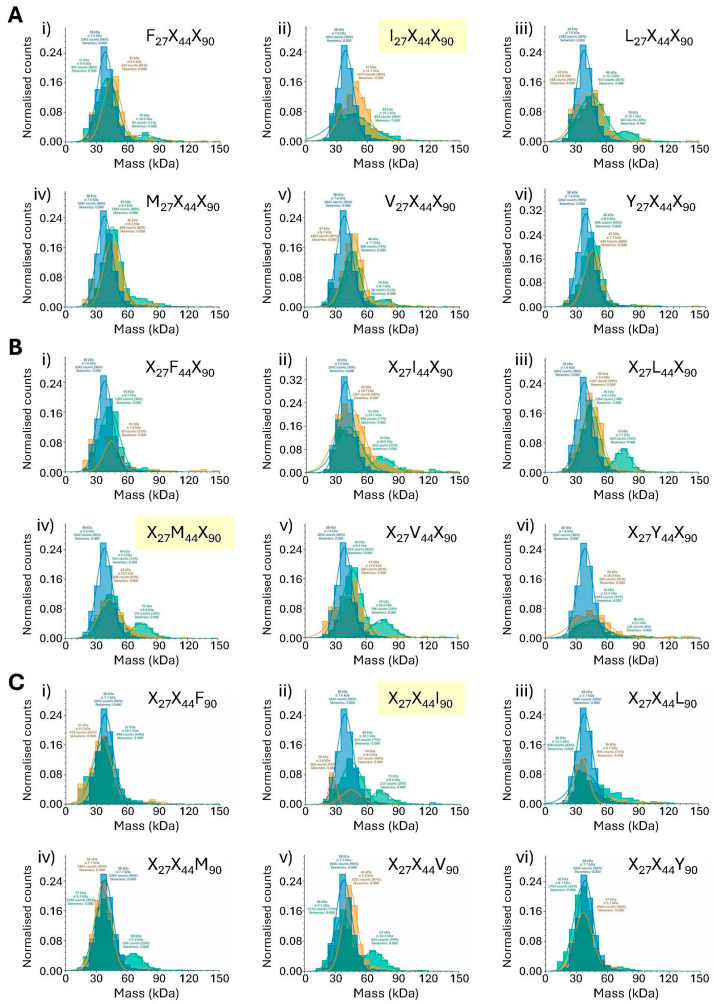
Mass photometry analysis of fixed-position SpyCatcher libraries interacting with native SpyTag. Three spectra are overlaid in each panel: green spectra represent the products of interaction between the relevant library and native SpyTag, while blue spectra represent native SpyTag and orange spectra the relevant SpyCatcher library for comparison. Each panel represents normalised counts of particles as a function of molecular mass (kDa). (**A**) SpyCatcher fixed position 27 libraries. (**B**) SpyCatcher fixed position 44 libraries. (**C**) SpyCatcher fixed position 90 libraries. (**i**) Fixed position = Phe. (**ii**) Fixed position = Ile. (**iii**) Fixed position = Leu. (**iv**) Fixed position = Met. (**v**) Fixed position = Val. (**vi**) Fixed position = Tyr. The identity of the SpyCatcher library in each spectrum is indicated, with those containing the native SpyCatcher highlighted in yellow. Count captions may be viewed via digital zoom or else are tabulated in Supplementary Table S3.

While it would be unrealistic to use mass photometry in a quantitative manner when examining mixtures of proteins (see below), we next sought to determine whether it could be used in a qualitative manner to examine the plasticity of the interaction between SpyCatcher at positions 27, 44 and 90 and native SpyTag. Accordingly, each of the 18 positionally fixed SpyCatcher protein libraries was incubated with native SpyCatcher in an 18:1 molar ratio and the interactions examined by mass photometry alongside the native SpyCatcher–SpyTag interaction as a reference (Figure 3).

With an equimolar SpyCatcher–SpyTag ratio, Figure 2 shows the previously revealed ~50% complexation under the conditions employed for mass photometry. By contrast, an 18:1 molar ratio of the SpyCatcher library–native SpyTag should approximate to a 0.5:1 ratio of any one individual SpyCatcher–SpyTag within that mixture. As such, simple extrapolation from Figure 2 to the data presented in Figure 3 would predict a peak of roughly half of 50%—i.e., approximately 25% complexation when native SpyCatcher (or an acceptable alternative) is present in a library. However, this assumes (a) that data is collected in the linear range of the interaction with respect to protein and peptide concentrations and (b) that all SpyCatcher library components are present in precisely equal concentrations. Although the codon distributions within individual libraries are good (Supplementary Figure S1), experimental distributions are never perfect, and further, it cannot be determined with certainty that even a precisely equal distribution of codons, albeit optimised for a given expression system, will necessarily result in a precisely equal distribution of expressed proteins. For these reasons, we contest that mass photometric analysis of libraries should be confined to a qualitative interpretation.

#### 3.4.1. Analysis of SpyCatcher Position 27 (Native SpyCatcher = Isoleucine)

It would be expected that any mass photometry peak denoting complex formation will be relatively small when detected from within a mixture of proteins. Nevertheless, an inspection of Figure 3(Ai–Avi) suggests that all SpyCatcher libraries fixed at position 27 except for Y_27_X_44_X_90_ showed some degree of covalent bond formation with native SpyTag as evidenced by the shoulder in the 70–90 kDa regions of the green histogram traces in Figure 3(Ai–Av). In some cases this shoulder was detected as a separate peak by the mass photometry software (Figure 3(Ai,Aiii,Av) where position 27 = Phe, Leu and Val, respectively) while in others (Figure 3(Aii,Aiv) where position 27 = Ile and Met, respectively) manual inspection of the spectra is required in order to see the shoulder that represents the interaction. Interestingly, this includes the only fixed position library to contain the native isoleucine residue at position 27 (Figure 3(Aii) and [25]). However, there is clearly a complete absence of any such shoulder in Figure 3(Avi), where position 27 is fixed as Tyr. This suggests that a wide variation in hydrophobic residues at position 27 of SpyCatcher can be tolerated in terms of binding the native SpyTag, although introduction of a polar hydroxyl group in the form of tyrosine is clearly not tolerated.

**Figure 4:**
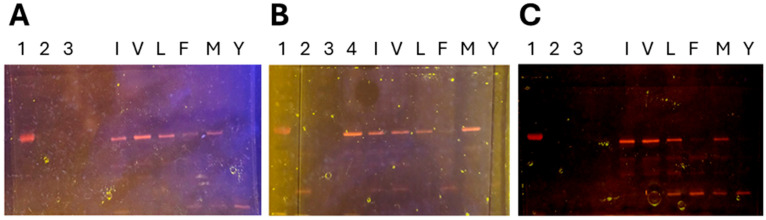
Polyacrylamide gel electrophoresis (SDS-PAGE, 4–12% gradient) of (**A**) fixed position 27 SpyCatcher libraries, (**B**) fixed position 44 SpyCatcher libraries, (**C**) fixed position 90 SpyCatcher libraries. Lanes: (1) MW marker (70 kDa); (2) SpyTag-mCherry fusion (30 kDa); (3) SpyCatcher-mNeonGreen fusion (42 kDa, not fluorescent); (4) (where present), Native SpyCatcher-mNeonGreen/Native SpyTag-mCherry complex; (I) fixed position = Ile; (V) fixed position = Val; (L) fixed position = Leu; (F) fixed position = Phe; (M) fixed position = Met; (Y) fixed position = Tyr. Gels were visualised by transillumination and digital photography. ST = SpyTag-mCherry fusion. SC-ST = SpyTag-mCherry/SpyCatcher-mNeonGreen complex.

To verify this analysis by alternate means, the fixed position 27 libraries were again incubated with native SpyTag at an 18:1 molar ratio and the products were examined by SDS-PAGE, noting that we had previously determined that while mNeonGreen fluorescence is eliminated under the denaturing conditions of SDS-PAGE, mCherry retains its chromophore (and thus fluorescence) following SDS-PAGE analysis (Figure 4A). Though surprising, this finding has also been reported elsewhere (e.g., [26]). The electrophoresis results confirmed the mass photometry analysis—namely, that of the six substitutions tested, any of the hydrophobic substitutions can be tolerated at position 27 of SpyCatcher, but substitution of Ile with Tyr eliminates the interaction—at least beyond levels detectable either by mass photometry or SDS-PAGE.

#### 3.4.2. Analysis of SpyCatcher Position 44 (Native SpyCatcher = Methionine)

The mass photometry analysis was repeated for the fixed position 44 SpyCatcher libraries. Results are illustrated in Figure 3(Bi–Bvi). Within this set of SpyCatcher libraries, there is a clear shoulder in the 70–90 kDa regions of the mass photometry spectra where position 44 is fixed as either Ile, Leu, Met (native) or Val (Figure 3(Bii–Bv), respectively). These findings were again confirmed by SDS-PAGE, where a clear band representing the covalent complex is visible in lanes I, V, L and M of Figure 4B. An additional faint band is also visible in lane F of Figure 4B, suggesting that weak complexation is possible when position 44 is fixed as Phe, whereas no convincing shoulder is apparent in Figure 3(Bi). Substitution of the native Met with Tyr at position 44 eliminates the SpyCatcher–SpyTag interaction as confirmed by both methodologies.

#### 3.4.3. Analysis of SpyCatcher Position 90 (Native SpyCatcher = Isoleucine)

Results of the interactions of fixed position 90 libraries with native SpyTag are illustrated in Figure 3(Ci–Cvi) (mass photometry) and Figure 4C (SDS-PAGE). Here, there are convincing peaks when position 90 is fixed as Ile (native), Met and Val (Figure 3(Cii,Civ,Cv), respectively) and a shoulder is evident where position 90 is fixed as Leu (Figure 3(Ciii)) whereas no shoulder is seen in the spectra in which position 90 is fixed as either Phe or Tyr (Figure 3(Ci,Cvi)). These results are again in agreement with the SDS-PAGE data, where clear bands representing the covalent SpyCatcher–SpyTag complex are seen Figure 4C lanes I, V, L and M, but there is no such band in lanes F and Y.

#### 3.4.4. SDS-PAGE Validates Mass Photometry Data

**Figure 5:**
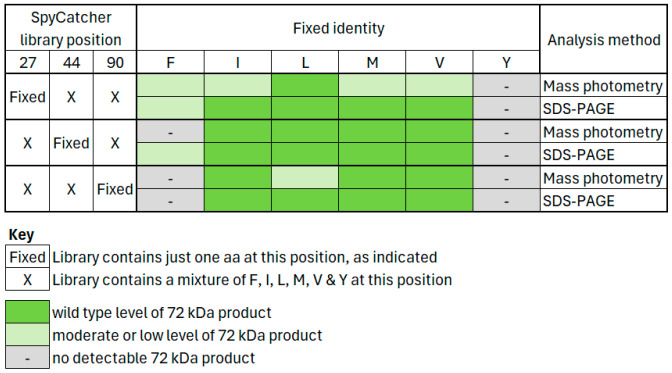
Summary of mass photometry (Figure 3) and SDS-PAGE (Figure 4) data for the interaction of SpyCatcher libraries with native SpyTag. Within each set of SpyCatcher libraries, wild type levels were assessed qualitatively by comparison between the one library that contains native SpyCatcher and the other libraries of that set in which native SpyCatcher is absent. Specifically, native SpyCatcher is present exclusively in libraries I_27_X_44_X_90_ (Figure 3, panel 3Aii and Figure 4A, lane “I”), X_27_M_44_X_90_ (Figure 3, panel 3Biv and Figure 4B, lane “M”) and X_27_X_44_I_90_ (Figure 3, panel 3Cii and Figure 4C, lane “I”).

The analyses of all fixed-position SpyCatcher libraries with respect to their complex formation with native SpyTag are summarised in Figure 5, which demonstrates clear agreement between the two methodologies, albeit that detection is slightly more sensitive using SDS-PAGE, when interactions are comparatively weak.

### 3.5. Application of SpyCatcher Library Data

The simplest interpretation of any set of positionally fixed libraries (whether biological or chemical) is to take the most effective substitution from one or more of each fixed position and to combine those substitutions together. In the current study, this would mean making individual SpyCatcher variants containing one or more of the substitutions identified in Section 3.4.1, Section 3.4.2 and Section 3.4.3. Each resulting novel SpyCatcher protein should, in theory, retain the ability to form a covalent bond with the native SpyTag peptide.

A simple, qualitative observation of the mass photometry data (Figure 3) reveals “good” peaks at 60–90 kDa in Figure 3(Aiii,Biii,Cv), where positions 27, 44 and 90 of SpyCatcher are fixed as Leu, Leu and Val, respectively. Accordingly, three proteins were selected for individual synthesis, containing one, two and three substitutions. The mass photometry peak when position 44 is substituted with Leu is particularly clear (Figure 3(Biii)). Therefore, Leu44 was selected as the single substitution for the first SpyCatcher variant (hereafter named “ILI”). The “best” substitution at position 27 was also identified as Leu (Figure 3(Aiii)) and so the second novel SpyCatcher protein was selected with substitutions Leu27 + Leu44 (hereafter “LLI”). Finally, examination of Figure 3C shows a particularly clear peak when position 90 is fixed as Val. Thus, the final variant SpyCatcher was selected to contain substitutions Leu27 + Leu44 + Val90 (hereafter “LLV”). Genes encoding each of the three proteins were created using overlap PCR, cloned as fusions to mNeonGreen and following sequence confirmation, were expressed and the resulting proteins isolated by IMAC.

### 3.6. Specificity of Native and Substituted SpyCatcher Proteins

**Figure 6:**
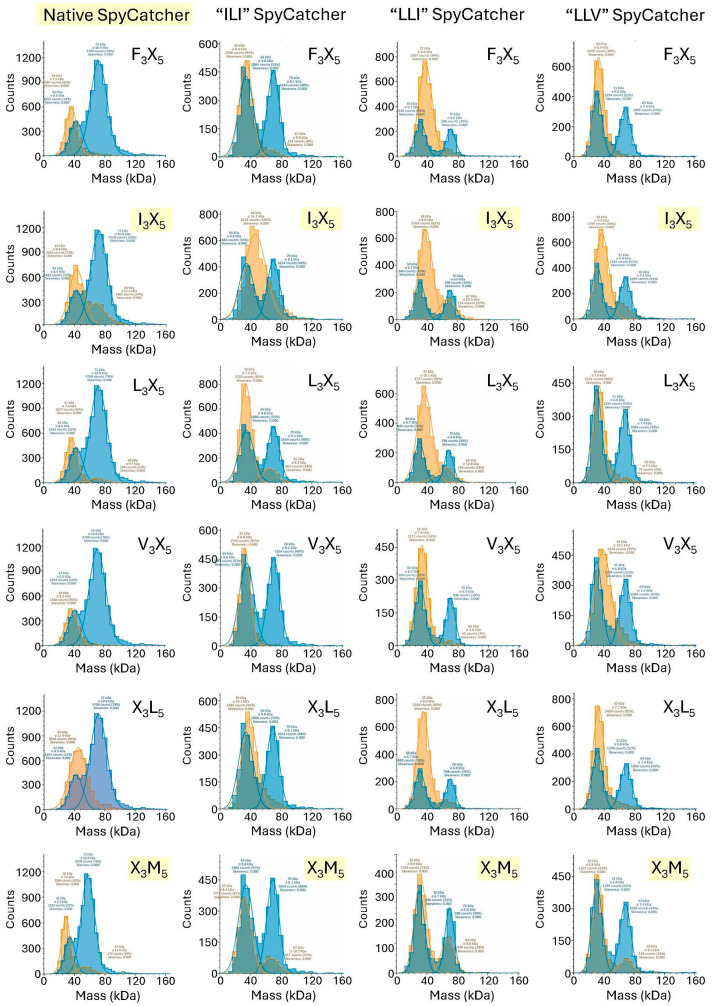
Mass photometry analysis of individual SpyCatcher proteins with selected fixed-position SpyTag libraries. Two spectra are overlaid in each panel. Orange spectra represent the products of interaction between the single SpyCatcher protein as indicated above each column with the indicated library. For comparison, blue spectra represent the interaction of the relevant individual SpyCatcher protein with native SpyTag. Each panel represents absolute counts of particles as a function of molecular mass (kDa). Note that in these spectra, green coloration is not a third spectrum but rather results from overlap of the individual blue and orange spectra. The identity of the SpyTag library in each spectrum is indicated, with those containing the native SpyTag highlighted in yellow. Count captions may be viewed via digital zoom or else are tabulated in Supplementary Table S4.

In any protein–peptide interaction, understanding the specificity of a reaction is at least as important as knowing the affinity. To examine the specificity of both the native and substituted SpyCatcher proteins, a preliminary PAGE analysis was undertaken with each of the SpyCatcher proteins (native SpyCatcher plus variants ILI, LLI and LLV) against both native SpyTag and all twelve SpyTag libraries. This preliminary analysis suggested that native SpyCatcher can react with SpyTag when position 3 of SpyTag is the native Ile, or else Leu, Phe or Val. Conversely, only native Met or alternatively, Leu are tolerated at position 5 of SpyTag (Figure S2). All four individual SpyCatcher proteins were then tested by mass photometry against both native SpyTag and the six SpyTag libraries that had given positive results by SDS-PAGE analysis—namely F_3_X_5_, I_3_X_5_, L_3_X_5_, V_3_X_5_, X_3_L_5_ and X_3_M_5_. Figure 6 shows the mass photometry spectrum of each single protein having reacted with native SpyTag, overlaid with the spectrum from interacting each SpyCatcher protein with the above SpyTag libraries (in a 1:6 ratio), for comparison.

**Figure 7:**
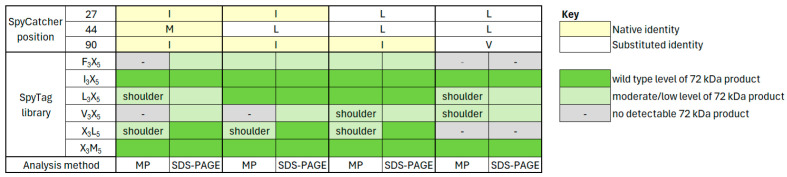
Summary of mass photometry (Figure 6) and SDS-PAGE (Figure S2) data for interactions between individual SpyCatcher proteins and SpyTag libraries. Wild-type levels were assessed qualitatively by comparison for each SpyCatcher protein between the libraries that contain native SpyTag and the other libraries in which native SpyTag is absent. Specifically, native SpyTag is present only in SpyTag library I_3_X_5_ (Figure 6, panels labelled I_3_X_5_ and Figure S2, Panel A, lanes “I”) and SpyTag library X_3_M_5_ (Figure 6, panels labelled X_3_M_5_ and Figure S2, Panel B, lanes “M”).

As can be seen in both Figure 6 and Figure S2, positional fixing has accurately predicted alternative substitutions in SpyCatcher. Each of the variant SpyCatcher proteins retains its ability to react with native SpyTag in a 1:1 reaction (Figure 6, blue traces), while the overlaid SpyTag library data (Figure 6, yellow traces) suggests the specificity of each SpyCatcher protein for variant SpyTags. The results from Figure 6 are summarised in Figure 7.

## 4. Discussion

This study aimed to explore the specificity of the SpyCatcher–SpyTag interaction via mass photometry analysis of positionally fixed libraries. Of course, there are many other means by which a protein–ligand or protein–peptide interaction can be measured even with respect to small libraries, but many require either additional labelling or the use of an antibody. Here, we present a new way to examine the interactions between small libraries of proteins and/or peptides in real time, without the need for any form of labelling (unless as in the current study, a boost in molecular mass is required). Inspection of Figure 5 and Figure 7 indicates that mass photometry, at least with respect to mixtures, is a little less sensitive than SDS-PAGE, but we suggest that this is not problematic—most importantly, in this study at least, mass photometry has not given a false positive. Moreover, the SpyCatcher–SpyTag interaction is a special case insomuch as the covalent, isopeptide bond formed from the SpyCatcher–SpyTag interaction gives an unusually simple means of verification. Most protein–peptide interactions are non-covalent and so verification by SDS-PAGE would be impossible, since the harsh conditions of boiling the sample and coating the protein with SDS would prevent complex visualisation. Conversely, mass photometry will show a stable interaction regardless of the types of bonding involved, since there is no denaturation involved, with the proviso that for non-covalent interactions the interaction should have a Kd within the working concentration range of mass photometry (i.e., ~200 nM or less). This limitation of analyte concentration results from the need for landing events to be separated spatially in order to allow for an appropriate “ratiometric” analysis of the images (see Figure 4 of [27]). However, where required, means have been developed to overcome the normal concentration limits, for example, by combining mass photometry with microfluidics [27] or by modifying the coverslip surface to lower the frequency of landing events [28].

In addition to protein–protein or protein–peptide interactions, mass photometry is a powerful technique that has been used to examine many other types of biomolecular interaction (e.g., those of nucleic acids [29], peptidiscs [30] and membrane proteins in various membrane-mimetic situations [31].) When considering single entities, mass photometry is both quantitative and can also be used to analyse binding kinetics [32]. As seen when examining either Figure 2, or the blue traces in Figure 3 and Figure 6, mass photometry is clearly quantitative, when studying the interaction of two purified entities. In contrast, we consider it overly ambitious to attempt any form of quantitation or when examining mixtures. To do so would require an absolutely even distribution of library components and this cannot be guaranteed even with the best methods of library preparation. Nevertheless, our data above do show clearly that a rapid, qualitative evaluation is possible. Specifically in two sets of experiments, we were able to evaluate 7776 possible interactions and the results/interpretation were substantiated by visualising the 1:1 complex formation of all of the elucidated SpyCatcher variants.

Of course, the interpretation of the lesser interactions when examining mass photometric spectra is somewhat subjective, but when engineering a new complex, a weak interaction is rarely required and as such, it is important not to place too great an emphasis on the “shoulders” in mass photometry spectra of libraries. In essence, mass photometry may give false negatives when analysing data, but from the data presented herein, we suggest that it is unlikely to offer a false positive result when screening libraries and as such a clear peak corresponding with the anticipated molecular mass of a complex is likely to correlate with a “good” substitution in a fixed position library.

It has been long established that the SpyCatcher–SpyTag interaction is very specific in terms of creating protein architectures. Orthogonality usually relies on using similar pairs from different sources, such as SnoopCatcher–SnoopTag (e.g., [5]). While the SpyCatcher–SpyTag pair has been engineered previously both by rational design and directed evolution, most previous engineering has focussed on improvement of the reaction time between SpyCatcher and SpyTag (SpyCatcher/Tag 002 and SpyCatcher/Tag 003 [8,9]). One previous study also examined the specificity of the interaction by substituting of Val and Ala for Phe at positions 75 and 92 of SpyCatcher and various hydrophobic residues at position 3 of SpyTag [33].

Meanwhile, our results suggest that substituting hydrophobic residues at positions 27, 44 and 90 of SpyCatcher and/or 3 and 5 of SpyTag is unlikely to elucidate orthogonal pairs of protein/peptide complexes, since we have demonstrated that native SpyTag can interact with several variants of the SpyCatcher protein (Figure 3 and Figure 4). Conversely, we have shown that substitution of any of these positions (either in SpyCatcher or SpyTag) with tyrosine completely eliminates the interaction between protein and peptide, which is consistent with previous reports that the hydrophobic pocket formed by these residues is important to create the required pKa of residues involved in isopeptide bond formation [3]. In contrast with our results, Liu et al. claim that SpyTag variant Tyr3 retains the ability to form a covalent complex with native SpyCatcher [33]. If it were possible to engineer a more polar interface between the protein and peptide without disrupting either gross protein structure or more importantly, the catalytic isopeptide bond formation between Asp7 of SpyTag and Lys31 of SpyCatcher (for which Glu77 of SpyCatcher is also essential [3,24]), then engineering completely orthogonal variants of the SpyCatcher–SpyTag pair might still be possible.

## 5. Conclusions

This study focused on the specificity of the SpyCatcher–SpyTag interaction through targeted hydrophobic substitutions in both SpyCatcher and corresponding SpyTag residues that have previously been identified as being important for isopeptide bond formation [3]. Interactions of variant SpyCatcher proteins and SpyTags were assessed both by conventional means (SDS-PAGE) and also by mass photometry. Our findings provide insight into the sequence–function relationship of the SpyTag–SpyCatcher interface and offer a potential framework for engineering modular covalent ligation tools with orthogonal specificity.

To the best of our knowledge, this is the first study in which mass photometry has been used to study the interactions of proteins from within mixtures or protein libraries and although herein the proteins of interest were fused to fluorescent proteins, that fusion was simply to allow both suitable molecular mass and also to facilitate verification by SDS-PAGE. We suggest that the current study has broader application. As far as mass photometry is concerned, no covalent interaction is required. As such, so long as the overall mass of the interaction product exceeds 30 kDa (herein, both partners as well as the final product exceeded 30 kDa), and the dissociation constant falls within a suitable range (high nM or better), interactions of components within small libraries can be elucidated, without the need for phage display or other commonly used means of screening small protein libraries.

## Data Availability

The original contributions presented in this study are included in the article/Supplementary Material. Further inquiries can be directed to the corresponding author(s).

## Supplementary Materials

The following supporting information can be downloaded at: https://www.mdpi.com/article/10.3390/biom15081183/s1, Table S1: Sequences of oligonucleotides used to construct SpyCatcher libraries; Table S2: Sequences of oligonucleotides used to construct SpyTag libraries; Table S3: Tabulation of counts data from main Figure 3; Table S4: Tabulation of counts data from main Figure 6; Figure S1: Composition of partially saturated SpyCatcher and SpyTag libraries; Figure S2: Polyacrylamide gel electrophoresis of fixed position SpyTag libraries with native and variant SpyCatcher proteins.

## Abbreviations

The following abbreviations are used in this manuscript:

IMAC	Immobilised Metal Affinity Chromatography
