# Supplementary material for: Engineering Protein–Peptide Interfaces via Combinatorial Mutagenesis and Mass Photometric Screening
Source: Biomolecules. 2025 Aug 18;15(8):1183. doi: 10.3390/biom15081183 (PMC12384687; doi:10.3390/biom15081183)
Supplement: Supplementary file 1 [file biomolecules-15-01183-s001.zip › biomolecules-3759444-supplementary.pdf]

biomolecules

MDPI

Table S1. Sequences of oligonucleotides used to construct SpyCatcher libraries. Libraries were constructed in 2 pieces: appropriate 27 Forward oligo(s) with 44 reverse oligo(s) and separately, 44 forward oligo(s) with 90 reverse oligo(s). Partially saturated positions (in final libraries) are indicated in red font. Required PCR products were joined by overlap PCR and then the final products each amplified with 27 forward and 90 reverse oligos. The resulting libraries were joined to appropriately amplified plasmid by Golden Gate seamless cloning.

Name	Sequence (5′-3′)
27 I Forward	GATAGTGCTACCCATATTAAATTCTCAAAACG
27 V Forward	GATAGTGCTACCCATGTGAAATTCTCAAAACG
27 L Forward	GATAGTGCTACCCATCTGAAATTCTCAAAACG
27 F Forward	GATAGTGCTACCCATTTTAAATTCTCAAAACG
27 M Forward	GATAGTGCTACCCATATGAAATTCTCAAAACG
27 Y Forward	GATAGTGCTACCCATTATAAATTCTCAAAACG
44 I Reverse	CACGCAACTCAATAGTTGCACCAGC
44 V Reverse	CACGCAACTCCACAGTTGCACCAGC
44 L Reverse	CACGCAACTCCAGAGTTGCACCAGC
44 F Reverse	CACGCAACTCAAAAGTTGCACCAGC
44 M Reverse	CACGCAACTCCATAGTTGCACCAGC
44 Y Reverse	CACGCAACTCATAAGTTGCACCAGC
44 I Forward	GCTGGTGCAACTATTGAGTTGCGTGAT
44 V Forward	GCTGGTGCAACTGTGGAGTTGCGTGAT
44 L Forward	GCTGGTGCAACTCTGGAGTTGCGTGAT
44 F Forward	GCTGGTGCAACTTTTGAGTTGCGTGAT
44 M Forward	GCTGGTGCAACTATGGAGTTGCGTGAT
44 Y Forward	GCTGGTGCAACTTATGAGTTGCGTGAT
90 I Reverse	GCTCATTAACTGTAAAGGTAATAGCAGTTGCTACC
90 V Reverse	GCTCATTAACTGTAAAGGTAACAGCAGTTGCTACC
90 L Reverse	GCTCATTAACTGTAAAGGTCAGAGCAGTTGCTACC
90 F Reverse	GCTCATTAACTGTAAAGGTAAAAGCAGTTGCTACC
90 M Reverse	GCTCATTAACTGTAAAGGTCATAGCAGTTGCTACC
90 Y Reverse	GCTCATTAACTGTAAAGGTATAAGCAGTTGCTACC
27 Forward	TTTTTTGGTCTCAGATAGTGCTACCCAT
90 Reverse	TTTTTTGGTCTCAGCTCATTAACTGTAAAGGT

Table S2. Sequences of oligonucleotides used to construct SpyTag libraries. Libraries were constructed by MAX randomisation, followed by Golden Gate seamless cloning into appropriately-amplified plasmid. Partially saturated positions (in final libraries) are indicated in red font. *Bsa*I restriction sites are underlined.

**Name**	**Sequence (5'-3')**
Template	GGCGTCTACNNNTACNNNGTGTGCCATCTA
Cassette start	CCATATTAAAGAGGAGAAATACTAGATG
Cassette end	GACGCCTACAAGCCGACGAAGGGT
MAX selection oligo, I_3	GCACACATT
MAX selection oligo, I_3V	GCACACGTG
MAX selection oligo, I_3L	GCACACCTG
MAX selection oligo, I_3F	GCACACTTT
MAX selection oligo, I_3M	GCACACATG
MAX selection oligo, I_3Y	GCACACTAT
MAX selection oligos, position M_5I	GTAATTGTA
MAX selection oligos, position M_5V	GTAGTGGTA
MAX selection oligos, position M_5L	GTACTGTA
MAX selection oligos, position M_5F	GTATTTGTA
MAX selection oligos, position M_5	GTAATGGTA
MAX selection oligos, position M_5Y	GTATATGTA
Forward primer	CCTTGTGGTCTCGCCATATTAAAGAGGAGAAATACT-AGATGGC
Reverse primer	CCTTGTGGTCTCACCCTTCGTCGGCTTGTAGG

Table S3 (cont.): Tabulation of counts data from main Figure 3. Colours are consistent with spectral captions as follows: **Green** - products of interaction between the relevant library and native SpyTag, **Blue** native SpyTag, **Orange** - SpyCatcher libraries as indicated.

**Library**	**Data**	**Library**	**Data**	**Library**	**Data**
**A i) $F_{27}X_{44}X_{90}$**	38 kDa<br>s 7.6 kDa<br>1042 counts (96%)<br>Skewness 0.000	**B i) $X_{27}F_{44}X_{90}$**	38 kDa<br>s 7.6 kDa<br>1042 counts (96%)<br>Skewness 0.000	**C i) $X_{27}X_{44}F_{90}$**	38 kDa<br>s 7.7 kDa<br>1045 counts (96%)<br>Skewness 0.000
	45 kDa<br>s 8.5 kDa<br>632 counts (81%)<br>Skewness 0.000		45 kDa<br>s 7.6 kDa<br>87 counts (51%)<br>Skewness 0.000		35 kDa<br>s 9.5 kDa<br>472 counts (92%)<br>Skewness 0.000
	41 kDa<br>s 9.4 kDa<br>492 counts (86%)<br>Skewness 0.000		43 kDa<br>s 9.5 kDa<br>1395 counts (96%)<br>Skewness 0.000		37 kDa<br>s 10.5 kDa<br>846 counts (94%)<br>Skewness 0.000
	76 kDa<br>s 10.9 kDa<br>63 counts (11%)<br>Skewness 0.000				

Table S3 (continued overleaf)

**A ii) $I_{27}X_{44}X_{90}$**	38 kDa<br>s 7.6 kDa<br>1042 counts (96%)<br>Skewness 0.000	**B ii) $X_{27}I_{44}X_{90}$**	38 kDa<br>s 7.6 kDa<br>1042 counts (96%)<br>Skewness 0.000	**C ii) $X_{27}X_{44}I_{90}$**	38 kDa<br>s 7.6 kDa<br>1042 counts (96%)<br>Skewness 0.000
	47 kDa<br>s 12.1 kDa<br>1079 counts (99%)<br>Skewness 0.000		42 kDa<br>s 13.7 kDa<br>1367 counts (98%)<br>Skewness 0.000		26 kDa<br>s 3.6 kDa<br>166 counts (42%)<br>Skewness 0.000
	44 kDa<br>s 19.1 kDa<br>893 counts (99%)<br>Skewness 0.000		41 kDa<br>s 13.1 kDa<br>904 counts (77%)<br>Skewness 0.000		44 kDa<br>s 9.0 kDa<br>212 counts (54%)<br>Skewness 0.000
			74 kDa<br>s 10.5 kDa<br>240 counts (21%)<br>Skewness 0.000		45 kDa<br>s 10.1 kDa<br>813 counts (75%)<br>Skewness 0.000
					73 kDa<br>s 8.6 kDa<br>215 counts (20%)<br>Skewness 0.000
**A iii) $L_{27}X_{44}X_{90}$**	38 kDa<br>s 7.6 kDa<br>1042 counts (96%)<br>Skewness 0.000	**B iii) $X_{27}L_{44}X_{90}$**	38 kDa<br>s 7.6 kDa<br>1042 counts (96%)<br>Skewness 0.000	**C iii) $X_{27}X_{44}L_{90}$**	38 kDa<br>s 7.7 kDa<br>1045 counts (96%)<br>Skewness 0.000
	43 kDa<br>s 13.0 kDa<br>538 counts (96%)<br>Skewness 0.000		45 kDa<br>s 9.3 kDa<br>1107 counts (94%)<br>Skewness 0.000		36 kDa<br>s 6.7 kDa<br>805 counts (74%)<br>Skewness 0.000
	46 kDa<br>s 13.2 kDa<br>917 counts (91%)<br>Skewness 0.000		45 kDa<br>s 8.5 kDa<br>1364 counts (74%)<br>Skewness 0.000		39 kDa<br>s 13.1 kDa<br>998 counts (83%)<br>Skewness 0.000
	78 kDa<br>s 10.1 kDa<br>163 counts (16%)<br>Skewness 0.000		76 kDa<br>s 7.7 kDa<br>440 counts (24%)<br>Skewness 0.000		
**A iv) $M_{27}X_{44}X_{90}$**	38 kDa<br>s 7.6 kDa<br>1042 counts (96%)<br>Skewness 0.000	**B iv) $X_{27}M_{44}X_{90}$**	38 kDa<br>s 7.6 kDa<br>1042 counts (96%)<br>Skewness 0.000	**C iv) $X_{27}X_{44}M_{90}$**	38 kDa<br>s 7.7 kDa<br>1045 counts (96%)<br>Skewness 0.000
	46 kDa<br>s 8.3 kDa<br>698 counts (82%)<br>Skewness 0.000		44 kDa<br>s 10.9 kDa<br>808 counts (92%)<br>Skewness 0.000		36 kDa<br>s 7.7 kDa<br>2905 counts (94%)<br>Skewness 0.000
	46 kDa<br>s 8.3 kDa<br>2307 counts (88%)<br>Skewness 0.000		44 kDa<br>s 9.3 kDa<br>524 counts (71%)<br>Skewness 0.000		37 kDa<br>s 9.2 kDa<br>2190 counts (83%)<br>Skewness 0.000

Table S3 (continued overleaf)

			75 kDa<br>s 8.8 kDa<br>174 counts (24%)<br>Skewness 0.000		66 kDa<br>s 9.3 kDa<br>536 counts (20%)<br>Skewness 0.000
**A v) $V_{27}X_{44}X_{90}$**	38 kDa<br>s 7.6 kDa<br>1042 counts (96%)<br>Skewness 0.000	**B v) $X_{27}V_{44}X_{90}$**	38 kDa<br>s 7.6 kDa<br>1042 counts (96%)<br>Skewness 0.000	**C v) $X_{27}X_{44}V_{90}$**	38 kDa<br>s 7.7 kDa<br>1045 counts (96%)<br>Skewness 0.000
	47 kDa<br>s 8.7 kDa<br>1405 counts (87%)<br>Skewness 0.000		43 kDa<br>s 13.9 kDa<br>360 counts (91%)<br>Skewness 0.000		44 kDa<br>s 7.2 kDa<br>1051 counts (87%)<br>Skewness 0.000
	46 kDa<br>s 7.7 kDa<br>368 counts (73%)<br>Skewness 0.000		46 kDa<br>s 9.3 kDa<br>2633 counts (81%)<br>Skewness 0.000		38 kDa<br>s 9.1 kDa<br>1576 counts (72%)<br>Skewness 0.000
	76 kDa<br>s 8.7 kDa<br>56 counts (11%)<br>Skewness 0.000		75 kDa<br>s 10.9 kDa<br>796 counts (24%)<br>Skewness 0.000		67 kDa<br>s 10.4 kDa<br>643 counts (29%)<br>Skewness 0.000
**A vi) $Y_{27}X_{44}X_{90}$**	38 kDa<br>s 7.6 kDa<br>1042 counts (96%)<br>Skewness 0.000	**B vi) $X_{27}Y_{44}X_{90}$**	38 kDa<br>s 7.6 kDa<br>1042 counts (96%)<br>Skewness 0.000	**C vi) $X_{27}X_{44}Y_{90}$**	38 kDa<br>s 7.7 kDa<br>1045 counts (96%)<br>Skewness 0.000
	45 kDa<br>s 7.7 kDa<br>630 counts (88%)<br>Skewness 0.000		43 kDa<br>s 16.0 kDa<br>299 counts (91%)<br>Skewness 0.000		37 kDa<br>s 9.1 kDa<br>2968 counts (96%)<br>Skewness 0.000
	44 kDa<br>s 8.4 kDa<br>846 counts (95%)<br>Skewness 0.000		44 kDa<br>s 13.4 kDa<br>1493 counts (91%)<br>Skewness 0.000		36 kDa<br>s 8.7 kDa<br>1702 counts (93%)<br>Skewness 0.000
			86 kDa<br>s 9.5 kDa<br>135 counts (8%)<br>Skewness 0.000		

Table S4 (cont.): Tabulation of counts data from main Figure 6. Colours are consistent with spectral captions as follows: **Orange** - products of interaction between the between the indicated single SpyCatcher protein with the indicated SpyTag library. **Blue** – Native SpyTag.

	**SpyCatcher protein**			
**SpyTag library**	**Native**	**ILI**	**LLI**	**LLV**
**F_3X_5**	42 kDa<br>s 8.5 kDa<br>1652 counts (22%)<br>Skewness 0.000	34 kDa<br>s 9.8 kDa<br>1884 counts (55%)<br>Skewness 0.000	30 kDa<br>s 6.7 kDa<br>840 counts (55%)<br>Skewness 0.000	31 kDa<br>s 6.8 kDa<br>1294 counts (52%)<br>Skewness 0.000
	72 kDa<br>s 10.9 kDa<br>5709 counts (78%)<br>Skewness 0.000	70 kDa<br>s 8.1 kDa<br>1654 counts (48%)<br>Skewness 0.000	70 kDa<br>s 6 kDa<br>598 counts (39%)<br>Skewness 0.000	69 kDa<br>s 7.4 kDa<br>1069 counts (43%)<br>Skewness 0.000
	36 kDa<br>s 7.5 kDa<br>985 counts (92%)<br>Skewness 0.000	34 kDa<br>s 8.4 kDa<br>1938 counts (93%)<br>Skewness 0.000	37 kDa<br>s 8.8 kDa<br>2967 counts (94%)<br>Skewness 0.000	33 kDa<br>s 6.9 kDa<br>2078 counts (90%)<br>Skewness 0.000
		67 kDa<br>s 8.8 kDa<br>155 counts (8%)<br>Skewness 0.000		
**I_3X_5**	42 kDa<br>s 8.5 kDa<br>1652 counts (22%)<br>Skewness 0.000	34 kDa<br>s 9.8 kDa<br>1884 counts (55%)<br>Skewness 0.000	30 kDa<br>s 6.7 kDa<br>840 counts (55%)<br>Skewness 0.000	31 kDa<br>s 6.8 kDa<br>1294 counts (52%)<br>Skewness 0.000
	72 kDa<br>s 10.9 kDa<br>5709 counts (78%)<br>Skewness 0.000	70 kDa<br>s 8.1 kDa<br>1654 counts (48%)<br>Skewness 0.000	70 kDa<br>s 6.0 kDa<br>598 counts (39%)<br>Skewness 0.000	69 kDa<br>s 7.4 kDa<br>1069 counts (43%)<br>Skewness 0.000
	43 kDa<br>s 9.8 kDa<br>3033 counts (75%)<br>Skewness 0.000	46 kDa<br>s 14. kDa<br>4131 counts (100%)<br>Skewness 0.000	38 kDa<br>s 9.0 kDa<br>2703 counts (81%)<br>Skewness 0.000	38 kDa<br>s 9.3 kDa<br>2799 counts (89%)<br>Skewness 0.000
	68 kDa<br>s 12.4 kDa<br>1481 counts (37%)<br>Skewness 0.000		67 kDa<br>s 10.5 kDa<br>722 counts (22%)<br>Skewness 0.000	
**L_3X_5**	42 kDa<br>s 8.5 kDa<br>1652 counts (22%)<br>Skewness 0.000	34 kDa<br>s 9.8 kDa<br>1884 counts (55%)<br>Skewness 0.000	30 kDa<br>s 6.7 kDa<br>840 counts (55%)<br>Skewness 0.000	31 kDa<br>s 6.8 kDa<br>1294 counts (52%)<br>Skewness 0.000
	72 kDa<br>s 10.9 kDa<br>5709 counts (78%)<br>Skewness 0.000	70 kDa<br>s 8.1 kDa<br>1654 counts (48%)<br>Skewness 0.000	70 kDa<br>s 6.0 kDa<br>598 counts (39%)<br>Skewness 0.000	69 kDa<br>s 7.4 kDa<br>1069 counts (43%)<br>Skewness 0.000

Table S4 (continued overleaf)

	37 kDa<br>s 7.6 kDa<br>1807 counts (89%)<br>Skewness 0.000	33 kDa<br>s 7.5 kDa<br>2720 counts (85%)<br>Skewness 0.000	37 kDa<br>s 10.1 kDa<br>2727 counts (92%)<br>Skewness 0.000	34 kDa<br>s 7.6 kDa<br>1501 counts (90%)<br>Skewness 0.000
	69 kDa<br>s 9.7 kDa<br>244 counts (12%)<br>Skewness 0.000	65 kDa<br>s 8.9 kDa<br>462 counts (14%)<br>Skewness 0.000	65 kDa<br>s 11.8 kDa<br>534 counts (18%)<br>Skewness 0.000	69 kDa<br>s 7.5 kDa<br>79 counts (5%)<br>Skewness 0.000
**V_3X_5**	42 kDa<br>s 8.5 kDa<br>1652 counts (22%)<br>Skewness 0.000	34 kDa<br>s 9.8 kDa<br>1884 counts (55%)<br>Skewness 0.000	30 kDa<br>s 6.7 kDa<br>840 counts (55%)<br>Skewness 0.000	31 kDa<br>s 6.8 kDa<br>1294 counts (52%)<br>Skewness 0.000
	72 kDa<br>s 10.9 kDa<br>5709 counts (78%)<br>Skewness 0.000	70 kDa<br>s 8.1 kDa<br>1654 counts (48%)<br>Skewness 0.000	70 kDa<br>s 6.0 kDa<br>598 counts (39%)<br>Skewness 0.000	69 kDa<br>s 7.4 kDa<br>1069 counts (43%)<br>Skewness 0.000
	38 kDa<br>s 8.2 kDa<br>1568 counts (95%)<br>Skewness 0.000	32 kDa<br>s 6.8 kDa<br>1524 counts (95%)<br>Skewness 0.000	33 kDa<br>s 7.6 kDa<br>1571 counts (94%)<br>Skewness 0.000	39 kDa<br>s 10.5 kDa<br>2244 counts (95%)<br>Skewness 0.000
			68 kDa<br>s 3.8 kDa<br>43 counts (3%)<br>Skewness 0.000	
**X_3L_5**	42 kDa<br>s 8.5 kDa<br>1652 counts (22%)<br>Skewness 0.000	34 kDa<br>s 9.8 kDa<br>1884 counts (55%)<br>Skewness 0.000	30 kDa<br>s 6.7 kDa<br>840 counts (55%)<br>Skewness 0.000	31 kDa<br>s 6.8 kDa<br>1294 counts (52%)<br>Skewness 0.000
	72 kDa<br>s 10.9 kDa<br>5709 counts (78%)<br>Skewness 0.000	70 kDa<br>s 8.1 kDa<br>1654 counts (48%)<br>Skewness 0.000	70 kDa<br>s 6.0 kDa<br>598 counts (39%)<br>Skewness 0.000	69 kDa<br>s 7.4 kDa<br>1069 counts (43%)<br>Skewness 0.000
	45 kDa<br>s 11.4 kDa<br>3910 counts (94%)<br>Skewness 0.000	39 kDa<br>s 10.1 kDa<br>2285 counts (95%)<br>Skewness 0.000	35 kDa<br>s 8.0 kDa<br>2563 counts (90%)<br>Skewness 0.000	33 kDa<br>s 7.2 kDa<br>2409 counts (91%)<br>Skewness 0.000
**X_3M_5**	42 kDa<br>s 8.5 kDa<br>1652 counts (22%)<br>Skewness 0.000	34 kDa<br>s 9.8 kDa<br>1884 counts (55%)<br>Skewness 0.000	30 kDa<br>s 6.7 kDa<br>840 counts (55%)<br>Skewness 0.000	31 kDa<br>s 6.8 kDa<br>1294 counts (52%)<br>Skewness 0.000
	72 kDa<br>s 10.9 kDa<br>5709 counts (78%)<br>Skewness 0.000	70 kDa<br>s 8.1 kDa<br>1654 counts (48%)<br>Skewness 0.000	70 kDa<br>s 6.0 kDa<br>598 counts (39%)<br>Skewness 0.000	69 kDa<br>s 7.4 kDa<br>1069 counts (43%)<br>Skewness 0.000
	36 kDa<br>s 7.0 kDa<br>2094 counts (83%)<br>Skewness 0.000	33 kDa<br>s 8.4 kDa<br>1377 counts (81%)<br>Skewness 0.000	32 kDa<br>s 8.0 kDa<br>1154 counts (73%)<br>Skewness 0.000	32 kDa<br>s 6.8 kDa<br>1381 counts (81%)<br>Skewness 0.000

Table S4 (continued overleaf)

	67 kDa	67 kDa	68 kDa	69 kDa
	σ 12.8 kDa	σ 10.3 kDa	σ 8.6 kDa	σ 8.6 kDa
	472 counts (19%)	357 counts (21%)	448 counts (28%)	243 counts (14%)
	Skewness 0.000	Skewness 0.000	Skewness 0.000	Skewness 0.000

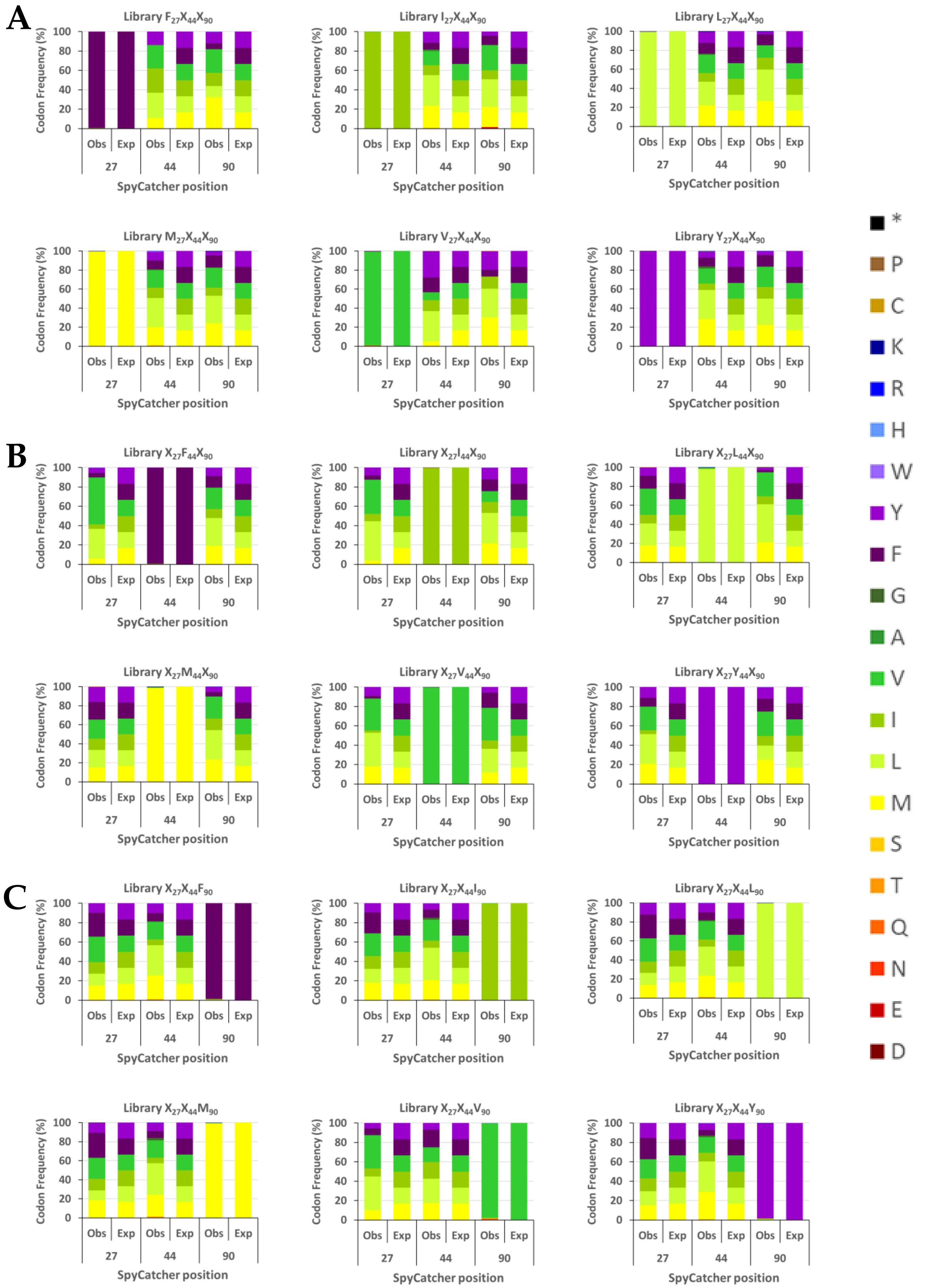
A
Library $F_{27}X_{44}X_{90}$
Library $I_{27}X_{44}X_{90}$
Library $L_{27}X_{44}X_{90}$
Library $M_{27}X_{44}X_{90}$
Library $V_{27}X_{44}X_{90}$
Library $Y_{27}X_{44}X_{90}$
B
Library $X_{27}F_{44}X_{90}$
Library $X_{27}I_{44}X_{90}$
Library $X_{27}L_{44}X_{90}$
Library $X_{27}M_{44}X_{90}$
Library $X_{27}V_{44}X_{90}$
Library $X_{27}Y_{44}X_{90}$
C
Library $X_{27}X_{44}F_{90}$
Library $X_{27}X_{44}I_{90}$
Library $X_{27}X_{44}L_{90}$
Library $X_{27}X_{44}M_{90}$
Library $X_{27}X_{44}V_{90}$
Library $X_{27}X_{44}Y_{90}$
Codon Frequency (%)
0
20
40
60
80
100
Obs
Exp
27
44
90
SpyCatcher position
*
P
C
K
R
H
W
Y
F
G
A
V
I
L
M
S
T
Q
N
E
D

D

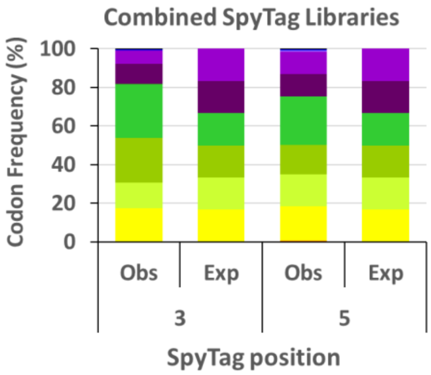

Figure S1. Composition of partially saturated SpyCatcher and SpyTag libraries.. NGS data was obtained and analysed as described in Materials and Methods. The identity of each library is indicated above each histogram. "Obs" = the observed codon frequency and "Exp" is the expected codon frequency for an ideal distribution **A-C:** SpyCatcher libraries, where: A) SpyCatcher fixed position 27 libraries. B) SpyCatcher fixed position 44 libraries. C) SpyCatcher fixed position 90 libraries. **D:** SpyTag libraries. In addition to individual Sanger sequencing (data not shown), each of the 6-member SpyTag libraries was combined and the resulting mixture analysed by NGS. Codon frequencies are as indicated in the labels alongside figures A-C.

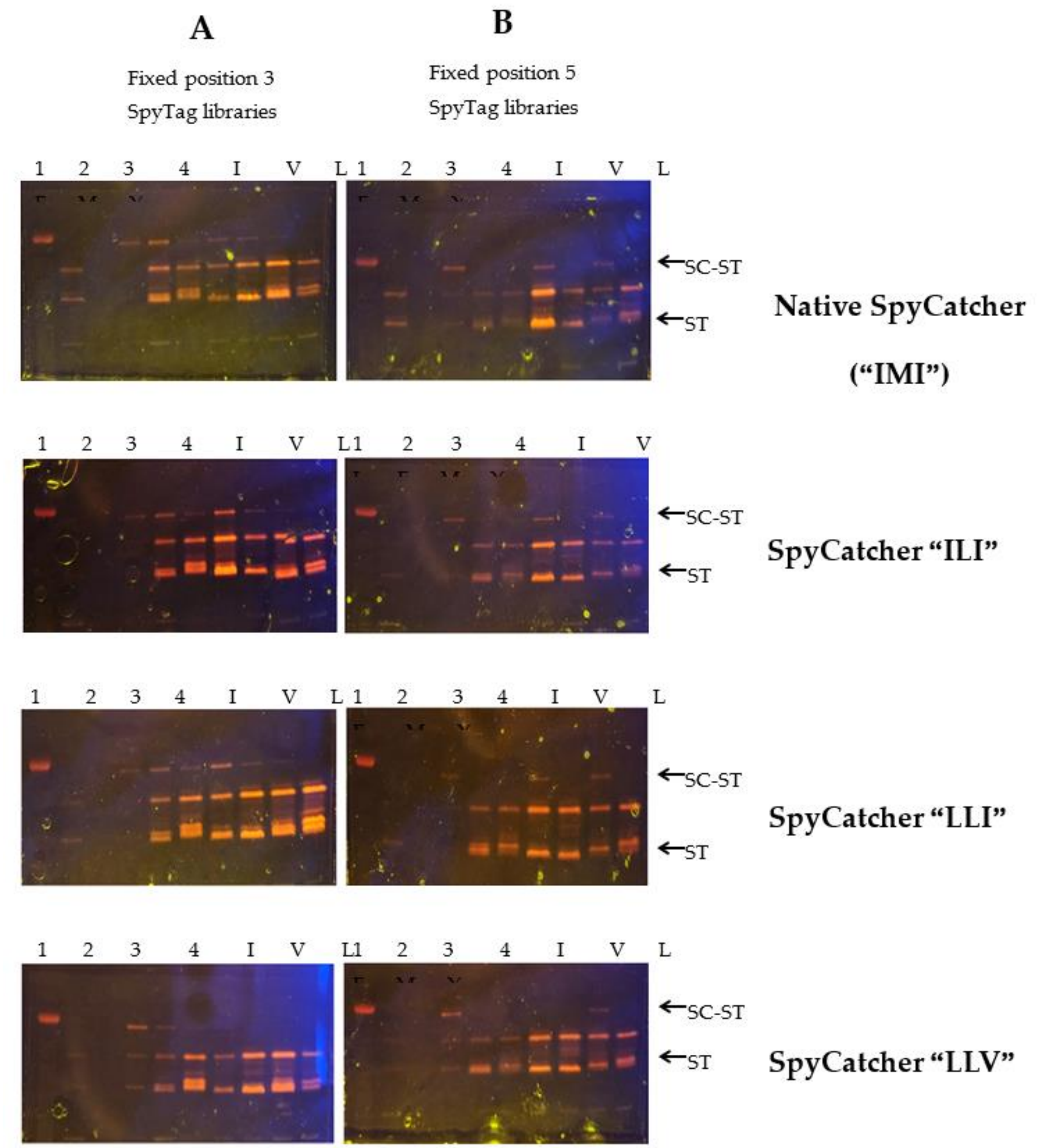

Figure S2. Polyacrylamide gel electrophoresis of fixed position SpyTag libraries with native and variant SpyCatcher proteins. SDS-PAGE, 4-12% gradient (A) fixed-position 3 SpyTag libraries, (B) fixed position 5 SpyTag libraries. Lanes: 1) MW marker (70 kDa); 2) SpyTag-mCherry fusion (30kDa); 3) SpyCatcher-mNeonGreen fusion (42kDa, not fluorescent); 4) Native SpyCatcher-mNeonGreen/Native SpyTag-mCherry complex; I) fixed position = Ile; V) fixed position = Val; L) fixed position = Leu; F) fixed position = Phe; M) fixed position = Met; Y) fixed position = Tyr. Gels were visualised by transillumination and digital photography. ST= SpyTag-mCherry fusion. SC-ST= SpyTag-mCherry/SpyCatcher-mNeonGreen complex. Note that in addition to the SpyTag-mCherry momomer, some self-complexation of this protein has occurred on storage (middle bands between ST and ST-SC).
